# Clinical and economic burden of community-acquired pneumonia in the Veterans Health Administration, 2011: a retrospective cohort study

**DOI:** 10.1007/s15010-015-0789-3

**Published:** 2015-05-17

**Authors:** John M. McLaughlin, Maribeth H. Johnson, Stephen A. Kagan, Stephanie L. Baer

**Affiliations:** Pfizer Vaccines, New York, NY USA; Charlie Norwood VA Medical Center, Augusta, GA USA; Georgia Regents University, Augusta, GA USA; PO BOX 113, Powell, OH 43065 USA

**Keywords:** Community-acquired pneumonia, Risk status, Risk factor, Burden of disease, Epidemiology, Veterans

## Abstract

**Purpose:**

The burden of community-acquired pneumonia (CAP) is not well described in the US Veterans Health Administration (VHA).

**Methods:**

CAP was defined as having a pneumonia diagnosis with evidence of chest X-ray, and no evidence of prior (90 days) hospitalization/long-term care. We calculated incidence rates of adult CAP occurring in inpatient or outpatient VHA settings in 2011. We also estimated the proportion of VHA CAP patients who were hospitalized, were readmitted within 30 days of hospital discharge, and died (any cause) in the year following diagnosis. Incremental costs during the 90 days following a CAP diagnosis were estimated from the perspective of the VHA.

**Results:**

In 2011, 34,101 Veterans developed CAP (35,380 episodes) over 7,739,757 VHA person-years. Median age of CAP patients was 65 years (95 % male). CAP incidence rates were higher for those aged ≥50 years. A majority of Veterans aged 50–64 (53 %) and ≥65 (66 %) years had ≥1 chronic medical (moderate risk) or immunocompromising (high risk) condition. Compared to those at low-risk (healthy), moderate- and high-risk Veterans were >3 and >6 times more likely to develop CAP, respectively. The percentage of CAP patients who were hospitalized was 45 %, ranging from 12 % (age 18–49, low risk) to 57 % (age ≥65, high risk). One-year all-cause mortality rates ranged from 1 % (age 18–49, low risk) to 36 % (age ≥65, high risk). Annual VHA medical expenditure related to CAP was estimated to be $750 million (M) ($415M for those aged ≥65 years).

**Conclusion:**

A focus on CAP prevention among older Veterans and those with comorbid or immunocompromising conditions is important.

**Electronic supplementary material:**

The online version of this article (doi:10.1007/s15010-015-0789-3) contains supplementary material, which is available to authorized users.

## Introduction

Community-acquired pneumonia (CAP) includes pneumonia transmitted outside of hospitals or extended-care facilities and is one of the most commonly diagnosed infectious diseases. There are over 100 pathogens known to cause CAP, including bacteria, viruses, and fungi. The most common bacterial pathogen of CAP is *Streptococcus (S.) pneumoniae,* which accounts for more than a third of all adult CAP [1], and has been shown to have mortality rates that are three times higher compared to other pathogens [2, 3].

An estimated 4–6 million (M) CAP cases occur annually in the United States, of which, roughly 20–25 % require hospitalization [4–6]. Overall mortality from CAP ranges 5–30 %, depending on the age and health of the patient. More severe cases of CAP are often associated with secondary bacteremia, where 30-day mortality is estimated to be as high as 20–48 % [3, 7–9]. Among all age groups, CAP is attributed to more than 10M physician visits, 64M days of restricted activity, and 600,000–1.1M hospitalizations each year in the United States [10–13], with an estimated US annual cost of more than $17 billion [7]. As the population ages, the burden of CAP is expected to increase. Although previous studies have highlighted the burden of CAP in several settings, less is known about the burden of CAP in the US Veterans Health Administration (VHA) population. Given that increased incidence of CAP has been shown to be related to being both older and male [14], the Veteran population is a unique and ideal population in which to measure both the clinical and economic burden of disease.

The VHA is the United States’ largest integrated healthcare system, providing care to 8.3 million Veterans every year, and is made up of more than 150 medical centers. These medical centers range in size and complexity from small, rural hospitals to tertiary care teaching facilities with academic medical center affiliations. In addition to VA hospitals and medical centers, the VHA system encompasses nearly 1400 outpatient clinics, nursing homes, inpatient and outpatient mental health facilities, home-based care services, and other health services.

## *S*tudy objective

We conducted a retrospective database analysis to calculate incidence rates of adult CAP occurring in inpatient or outpatient VHA facilities in 2011. We also estimated the proportion of VHA CAP patients who were hospitalized, were readmitted within 30 days of hospital discharge (if hospitalized), and died (any cause) in the year following diagnosis. Incremental costs that were accrued during the 90 days following a CAP diagnosis were estimated from the perspective of the VHA. Results describe both the overall and stratified (by age and risk status) burden of illness associated with CAP.

## Methodology

### Study population

We retrospectively identified patients aged ≥18 years in the VHA Corporate Data Warehouse (CDW). CDW is composed of data from the entire VHA system, including both clinical and administrative data. Previous studies have noted that VHA users tend to be poorer, older, less educated, more likely to be unemployed or underemployed, more likely to be African American, and more likely to be in poorer health than either the general population or Veterans who do not use the VHA system [15, 16]. To receive care in the VHA system, Veterans must qualify for health benefits based on their history of military service, current financial resources, and level of ‘service connectedness’. Medical conditions or disabilities are considered (partially or fully) ‘service connected’ if the illness or injury is determined to have occurred while in (or soon after) military service, have been a pre-existing condition aggravated by military service, or be related to a specific exposure while in service. We obtained study approval from the Veterans Affairs institutional review board and all data were de-identified prior to analysis.

### Identification of community-acquired pneumonia episodes

Pneumonia episodes began on the diagnosis (inpatient or outpatient) date of the first medical claim with any diagnosis of pneumonia (ICD9: 480–486, 487.0) from an inpatient or outpatient setting from January 1 through December 31 of 2011. To be included, pneumonia episodes also had to have a medical claim with a procedure code for a chest X-ray (CPT code 71010–71035; ICD9-CM 87.4x) within 14 days before or after the initial diagnosis of pneumonia. To characterize episodes as community acquired, we excluded those with any claim for mechanical ventilation, long-term care, hospitalization, pneumoconiosis, or wound care therapy in the 90 days prior to diagnosis [17]. CAP episodes with claims for treatment in the outpatient setting (including dialysis and chemotherapy infusion centers) in the 90 days prior to diagnosis were not excluded. CAP episodes ended on the date of the last observed medical claim with diagnosis for pneumonia that was followed by a minimum period of 90 days during which no medical claims with diagnoses of pneumonia were observed (“clean period”). This 90-day period was pre-specified as sufficient time to distinguish distinct CAP episodes and has been described previously [18]. An individual patient could have >1 CAP episode during the study period. Episodes that began at a late enough date that 90 days could not be observed between the last medical claim with a pneumonia diagnosis and the end of the study were included and censored at December 31, 2011. Episodes in which the patient died within the 90 days following the CAP episode were also included and were censored at the date of death. To determine whether or not a patient was hospitalized for CAP, we evaluated whether any inpatient claims (versus outpatient only) for CAP were present on the day of diagnosis or in the 30 days following the diagnosis.

### Vital status

Mortality data were available from several sources within the VHA. The VHA Vital Status File includes mortality data from the inpatient Patient Treatment File, the Beneficiary Identification & Records Locator System, Medicare, and the Social Security Administration death file. Data quality and accuracy from the combined data sources within the Vital Status File have been shown to be nearly as complete as the National Death Index.

### Risk status

Patients were categorized into risk groups (low, moderate, or high) based on the presence of underlying immunocompromising or chronic medical conditions [19–21]. *High risk* was defined as having any immunocompromising condition including: human immunodeficiency virus/acquired immune deficiency syndrome (HIV/AIDS); leukemia, lymphoma, Hodgkin’s Disease; generalized malignancy, excluding skin cancer; multiple myeloma; diseases requiring treatment with immunosuppressive drugs including long-term corticosteroids or radiation therapy; nephrotic syndrome; chronic renal failure, including end-stage renal disease (ESRD); organ transplantation; cochlear implants, CSF leaks; sickle cell disease, functional or anatomic asplenia; and immune deficiency and other conditions consistent with an immunocompromised state [19–21]. *Moderate risk* was defined as the absence of immunocompromising conditions but the presence of ≥1 chronic medical condition including: chronic heart disease, heart failure, cardiomyopathy, coronary artery disease; diabetes; chronic lung disease, COPD, asthma; liver disease or alcoholism [19–21]. *Low risk* was defined as immunocompetent without any of the chronic medical conditions listed above [19–21].

### Economic data

Economic data were extracted from the CDW Health Economic Research Center (HERC) to estimate the incremental, average cost associated with developing CAP from the perspective of the VHA. The VHA is funded primarily by a Congressional appropriation; therefore, its utilization data do not assign costs or charges to specific patient encounters. To estimate the cost of medical services provided to VHA patients, we used average cost methods developed by HERC. Detailed descriptions of these methods, modeling assumptions, and comparisons to alternative costing methods have been published previously [22, 23]. The average cost method is used to model the cost of VHA services based on similar, hypothetical Medicare payments. HERC inpatient estimates represent the regionally adjusted, national average cost of similar Medicare hospital stays of Veterans in non-VA hospitals given VHA information about the Diagnosis Related Group, overall length of stay, and days in intensive care. HERC outpatient estimates represent the hypothetical, average Medicare reimbursement value, given the Current Procedure and Terminology codes assigned to a particular VHA outpatient visit.

### Statistical analysis

Incidence rates (IRs) were constructed by dividing the number of CAP episodes that occurred in the VHA in 2011 by the number of person-years (PYs) accrued in the VHA-enrolled population in that same year by age and risk groups. Incidence rate ratios (IRRs) and 95 % confidence intervals (CIs) were constructed by comparing IRs between various age and risk groups.

Among those who developed CAP, multivariable models were constructed to assess the effect of underlying risk status on the likelihood of initial hospitalization, 30-day readmission (among those who were hospitalized), and death, accounting for age and other sociodemographic factors. Because hospitalization for CAP was relatively common (45 %) and because logistic regression events are not well suited to predict common (> 10 %) events, log binomial regression models were used to estimate relative risk of hospitalization. To assess the relationship between patient-level factors and the proportion of patients who (1) were admitted to the hospital for CAP and then readmitted within 30 days of discharge for any cause and (2) died in the same year as the index CAP episode, logistic regression analyses were used.

For both log binomial and logistic regression analyses, the Likelihood Ratio (LR) Chi square test was used to determine improved statistical fit using a *p* value <.05 as significant model improvement. Additionally, for all models, each justifiable interaction term was tested for statistical significance. Confounding was defined as a variable that changed the estimated relative risk or odds ratio (OR) of a variable already in the model by ≥10–15 %. Any significant interaction terms or appreciable confounders remained in the final model. Because this was an exploratory burden of disease analysis, no adjustments for multiple comparisons were made. Thus, a two-sided alpha of .05 was used for all analyses.

Patients who were missing valid HERC data were excluded from cost analyses. For each patient with valid HERC data, estimates of inpatient and outpatient economic costs for each clinical encounter in the VHA system were developed for each primary episode of CAP and then aggregated into per-patient, per-month (PPPM) costs for three time periods: (1) the 180 days prior to the development of CAP, (2) the 90 days following the diagnosis of CAP—including the diagnosis date (i.e., “CAP episode”), and (3) the 180 days after the (90-day) CAP episode. Given the skewed (non-Gaussian) nature of health care cost data, differences between costs in the 180-day pre-CAP episode (baseline) periods were compared to the costs of both (1) the 90-day CAP episode and (2) the 180-days following the (90 day) CAP episode using non-parametric Wilcoxon sign-rank tests. Finally, we assessed predictors of (90-day) CAP episode costs using a non-parametric quantile regression model.

## Results

### Patient characteristics and incidence of community-acquired pneumonia

**Fig. 1:**
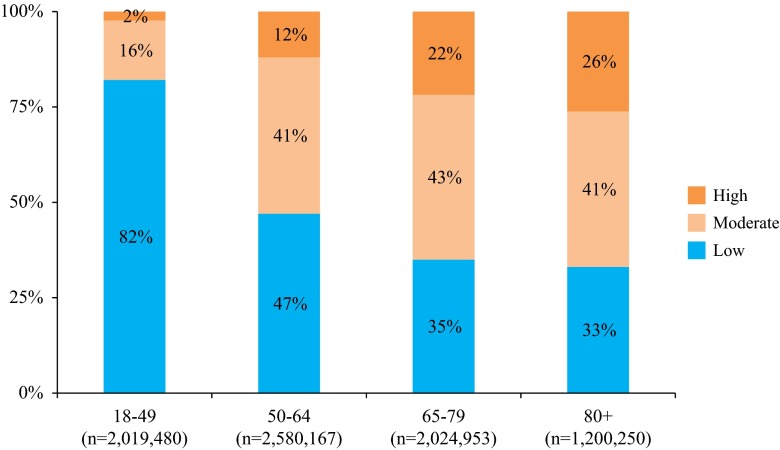
Proportion of all adults in the Veterans Health Administration at low, moderate, and high risk for developing community-acquired pneumonia by age group, 2011 (*n* = 7,824,850). Low risk was defined as immunocompetent without chronic medical conditions. Moderate risk was defined as immunocompetent with ≥1 chronic medical condition. High risk was defined as immunocompromised

In 2011, 7,824,850 patients were enrolled in the VHA. Of which, 26, 33, 26, and 15 % were aged 18–49, 50–64, 65–79, and ≥80 years, respectively. A majority of VHA patients aged 50–64 (53 %) and aged ≥65 years (66 %) had ≥1 chronic medical or immunocompromising condition that put them at increased risk for developing CAP. Twenty-three percent of all VHA patients aged ≥65 years had an immunocompromising condition (Fig. 1).

**Fig. 2:**
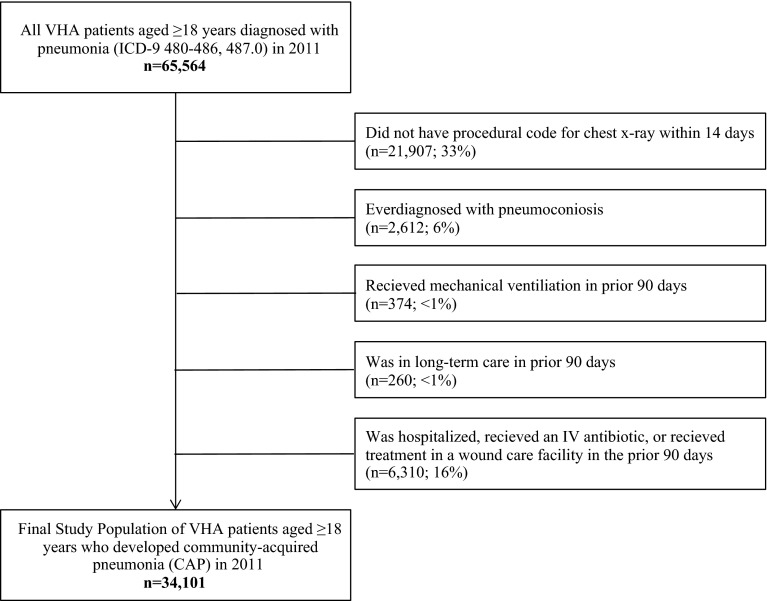
Selection criteria

In 2011, after applying selection criteria (Fig. 2), 34,101 adult VHA patients developed CAP (35,380 episodes) over 7,739,757 VHA person-years. Three percent of CAP patients had >1 CAP episode during 2011. Median age of CAP patients was 65 years (95 % male). Of those who developed CAP, most were white race (74 %) and non-Hispanic (97 %). Roughly half were married (47 %) and service connected (45 %). A majority were exempt from copayment (82 %), and had some type of supplemental insurance (63 %) (eTable 1). Age was strongly related to the proportion of patients who had supplemental coverage, as patients who were Medicare age-eligible (≥65 years) were much more likely to have supplemental coverage (18–49: 30 %; 50–64: 40 %; 65–79: 86 %; ≥80: 87 %; *p* for interaction <.001).

**Fig. 3:**
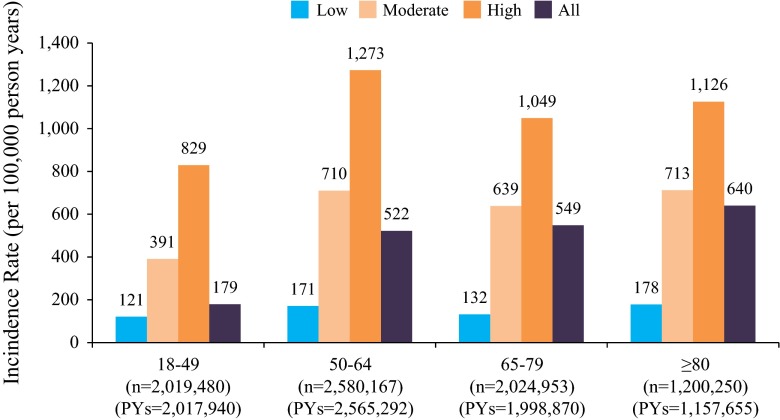
Incidence rate (per 100,000 person-years) of community-acquired pneumonia (CAP) by age and risk of developing cap in the Veterans Health Administration, 2011 (episodes = 35,380, *n* = 7,824,850; PYs = 7,739,757). *PYs* are person-years. Low risk was defined as immunocompetent without chronic medical conditions. Moderate risk was defined as immunocompetent with ≥1 chronic medical condition. High risk was defined as immunocompromised

CAP incidence rates were higher for those aged ≥50 vs 18–49 years. Incidence rates among those aged 50–64 and ≥65 years were similar. Compared to those at low risk, moderate- and high-risk patients were >3 and >6 times more likely to develop CAP, respectively (Fig. 3; Table 1). In addition, compared to patients without comorbid or immunocompromising conditions, patients with a history of diabetes, dementia, or chronic heart, cerebrovascular, lung, or liver disease were 3–14 times more likely to develop CAP, depending on the specific comorbid illness and age of the patient (Table 1). Patients with a history of heart failure (11–14 times more likely) or COPD (7–12 times more likely) were especially more likely to develop CAP across all age groups (Table 1).

**Table 1:** Incidence rate ratios (IRR) and 95 % confidence intervals (CI) for developing community-acquired pneumonia (CAP) by age group and risk of developing CAP in the Veterans Health Administration, 2011 (episodes = 35,380, *n* = 7,824,850; PYs = 7,739,757)

Risk factor	Age group, IRR (95 % CI)			
	18–49 years *n* = 2,019,480 PYs = 2,017,940	50–64 years *n* = 2,580,167 PYs = 2,565,292	65–79 years *n* = 2,024,953 PYs = 1,998,870	≥80 years *n* = 1,200,250 PYs = 1,157,655
Aggregate risk for CAP*				
Low	1.00	1.00	1.00	1.00
Moderate	3.24 (3.12, 3.35)	4.15 (4.11, 4.19)	4.83 (4.79, 4.87)	4.00 (3.95, 4.05)
High	6.86 (6.76, 6.97)	7.44 (7.39, 7.49)	7.94 (7.97, 8.01)	6.32 (6.24, 6.40)
Select comorbidities^†^				
Heart Failure	11.6 (11.4, 11.8)	11.4 (11.3, 11.4)	14.0 (13.9, 14.1)	11.1 (11.0, 11.1)
Coronary artery disease	4.55 (4.40, 4.69)	5.42 (5.38, 5.46)	6.09 (6.05, 6.13)	4.78 (4.73, 4.82)
Stroke	4.50 (4.23, 4.77)	5.78 (5.72, 5.84)	7.86 (7.81, 7.91)	7.57 (7.52, 7.63)
Diabetes	3.42 (3.32, 3.53)	4.35 (4.31, 4.38)	5.41 (5.37, 5.45)	4.77 (4.72, 4.82)
COPD	7.57 (7.45, 7.68)	9.54 (9.50, 9.57)	12.2 (12.2, 12.2)	9.60 (9.55, 9.65)
Asthma	4.49 (4.39, 4.59)	6.79 (6.73, 6.84)	9.08 (9.02, 9.14)	7.66 (7.57, 7.74)
Chronic liver disease	4.43 (4.26, 4.59)	6.27 (6.22, 6.32)	9.30 (9.21, 9.38)	10.2 (10.1, 10.4)
Alcoholism	3.01 (2.91, 3.11)	5.26 (5.22, 5.30)	8.78 (8.71, 8.84)	8.79 (8.66, 8.92)
Dementia	3.52 (3.25, 3.79)	6.46 (6.36, 6.56)	8.53 (8.46, 8.60)	7.51 (7.46, 7.56)

*PYs* Person-years and *COPD* chronic obstructive pulmonary disorder

* Low risk was defined as immunocompetent without chronic medical conditions. Moderate risk was defined as immunocompetent with ≥1 chronic medical condition. High risk was defined as immunocompromised

^†^For each comorbidity category, low-risk patients (i.e., immunocompetent without chronic medical conditions) are the reference category. Each comorbidity category was defined as having any claim for the selected underlying comorbid condition, regardless of history of other comorbid or immunocompromising conditions

### Clinical outcomes of community-acquired pneumonia

Older CAP patients and those at moderate or high risk were more likely to be hospitalized and had higher mortality rates. The percentage of all adult CAP patients who were hospitalized was 45 %, ranging from 12 % (age 18–49 at low risk) to 57 % (age ≥65 at high risk). Among those who were hospitalized, average length of stay was 8 days (median 5 days) and 16 % were readmitted (any cause) within 30 days of discharge. Among CAP patients, same-year, all-cause mortality rates ranged from 1 % (age 18–49 at low risk) to 36 % (age ≥65 at high risk) (Table 2).

**Table 2:** Proportion of community-acquired pneumonia (CAP) Patients who were hospitalized for CAP, readmitted (any cause) within 30-days of hospital discharge, or died in the same year as the CAP event by risk status and age Group, 2011 (*n* = 34,101)

Risk of CAP*	Low *n* (%)			Moderate *n* (%)			High *n* (%)		
	Hospitalized	30-Day all-cause readmission	Died	Hospitalized	30-Day all-cause readmission	Died	Hospitalized	30-Day all-cause readmission	Died
Age group									
18–49 (*n* = 3,557)	234 (12)	23 (10)	21 (1)	315 (26)	40 (13)	32 (3)	159 (42)	30 (19)	37 (10)
50–64 (*n* = 12,962)	409 (20)	32 (8)	85 (4)	3,072 (43)	475 (15)	603 (8)	2,019 (55)	409 (20)	978 (27)
65–79 (*n* = 10,515)	230 (25)	24 (10)	82 (9)	2,451 (46)	348 (14)	786 (15)	2,371 (55)	433 (18)	1,353 (32)
≥80 (*n* = 7,067)	302 (45)	40 (13)	189 (28)	1,798 (56)	245 (14)	1,010 (31)	1,905 (60)	278 (15)	1,309 (41)
All Ages	1,175 (21)	119 (10)	377 (7)	7,636 (45)	1,108 (15)	2,431 (14)	6,454 (56)	1,150 (18)	3,677 (32)

* Low risk was defined as immunocompetent without chronic medical conditions. Moderate risk was defined as immunocompetent with ≥1 chronic medical condition. High risk was defined as immunocompromised

Of the VHA patients who developed CAP in 2011, the vast majority aged 50–64 (84 %) and ≥65 (91 %) years had some type of underlying immunocompromising or chronic medical condition (eTable 2). Immunocompromising conditions were composed mainly of chronic renal disease or solid organ cancer. Prevalent chronic medical conditions among those who developed CAP included history of heart failure, coronary artery disease, diabetes, and COPD. Although not common among those aged ≥65 years, among those aged 50–64 years, chronic liver disease and alcoholism were prevalent among those who developed CAP in the VHA (eTable 2).

Results of multivariable modeling showed that even after adjustment for age and other sociodemographic factors, compared to patients at low risk, patients at moderate risk were 1.85 (95 % CI 1.71, 1.98) times more likely to be hospitalized for CAP, 1.47 (95 % CI 1.19, 1.80) times more likely to be readmitted (any cause) within 30 days if they were hospitalized, and 1.51 (95 % CI 1.34, 1.71) times as likely to die in the same year of the CAP episode. High-risk patients (compared to low-risk patients) were 2.11 (95 % CI 1.96, 2.27) times more likely to be hospitalized for CAP, 1.90 (95 %CI 1.54, 2.33) times more likely to be readmitted (any cause) within 30 days if they were hospitalized, and 3.75 (95 % CI 3.32, 4.23) times as likely to die in the same year of the CAP episode (eTable 3). Other factors associated with hospitalization for CAP (*p* < .05) included increasing age, being unmarried, not having service connection, not having supplemental health insurance, and being exempt from copayment. Another factor associated with 30-day readmission after a CAP hospitalization (any cause) (*p* < .05) was not having supplemental health insurance. Other factors associated with mortality in the same year of developing CAP (*p* < .05) included increasing age, not having supplemental health insurance, and being hospitalized for the CAP episode (eTable 3).

### Economic impact of community-acquired pneumonia

**Fig. 4:**
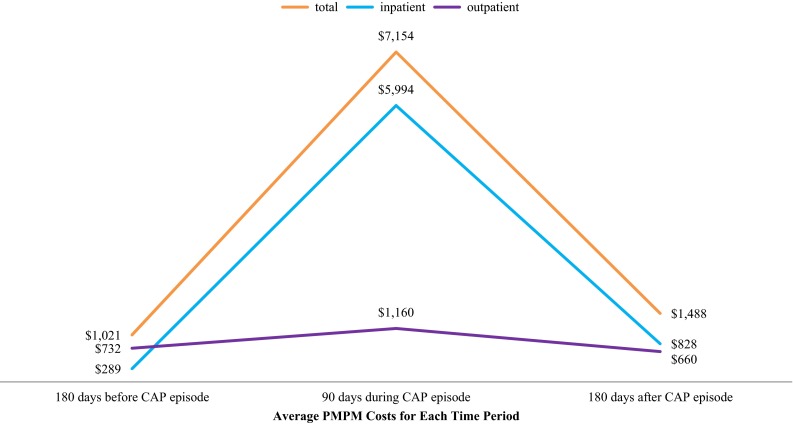
Average per-patient, per-month (pppm) costs before, during, and after the development of community-acquired pneumonia (CAP) among Veterans Health Administration-eligible persons who CAP, 2011 (*n* = 34,049). 52 of 34,101 patients were missing valid economic data from the HERC dataset. The “CAP episode” was defined as the 90 days following the diagnosis of CAP—including the diagnosis date. Data were adjusted to account for local variations in costs

Among all VHA patients who developed CAP in 2011, 52 (<0.2 %) patients were missing valid HERC cost data and were excluded from cost analyses. Average total per-patient, per-month (PPPM) costs increased from $1,020 (median $381) during the 180-day period before the CAP episode to $7,154 (median $3,174) during the 90-day CAP episode period (*p* < .0001) (Fig. 4). In the 180 days following the 90-day CAP episode, PPPM costs declined to $1488 (median $347), but average PPPM costs (though not median PPPM costs) still remained higher than the pre-CAP episode period. However, if patients without adequate post-CAP episode follow-up data were excluded (i.e., patients who died during 2011), both mean and median PPPM costs during the 180-day post-CAP period were higher (median $1,401 vs $410; *p* < .0001) than that in the 180-day, pre-CAP period. These trends were consistent across all age/risk strata and were driven primarily by increases in the cost of inpatient care (eFigure 1). If we assume that these increases in cost from the 180-day, pre-CAP baseline period stem from CAP- and CAP sequelae-related care, the excess economic burden attributable to each CAP episode in the (1) 90 days during the CAP episode and (2) 180 days following the (90-day) CAP episode was $18,399 and $2,804, respectively. Thus, for the 35,380 CAP episodes that occurred in 2011, the excess economic burden was estimated to be $750.2 M ($415.1 M for those aged ≥ 65 years) during the 90-day CAP episode period and the period of 180 days following the episode—driven mainly by an estimated $719.9 M in additional inpatient expenditures (eTable 4). Final quantile regression modeling showed that increasing age, black race (compared to white race), being unmarried, having 50–100 % service connection, not having supplemental insurance, having underlying comorbid or immunocompromising conditions, and being hospitalized were all related to significantly higher median CAP episode VHA costs (eTable 5).

## Discussion

Community-acquired pneumonia remains a significant health problem in the VHA, with >35,000 episodes (according to our definition) occurring within the VHA system in 2011 alone. These CAP episodes accounted for an estimated $750 M ($415 M for those aged ≥ 65 years) worth of additional VHA annual medical costs—the large majority of which ($720 M; 96 %) were due to additional expenditures related to hospitalization and other inpatient services. It is also worth noting that, among patients who survived a CAP episode and had sufficient post-episode follow-up, patient health care costs were not only significantly higher during the CAP episode, but they also remained higher during the 6 months following the (90-day) CAP episode as well.

This significant burden of CAP persists despite the fact that VHA patients have pneumococcal polysaccharide vaccination coverage that is notably better than both (1) the general, non-VA population and (2) Veterans who do not participate in VHA [24]. At the time of this study, only 23-valent pneumococcal polysaccharide vaccine was approved for routine use in adults. Since this study was conducted, however, 13-valent pneumococcal conjugate vaccine (PCV13) was approved for use in adults aged ≥50 years on December 30, 2011 [25], and subsequently recommended for routine use in all adults aged ≥65 years by ACIP in September 2014 [26]. CDC estimated that “…10 % of community-acquired pneumonia cases in adults aged ≥65 years are caused by PCV13 serotypes and are potentially preventable with the use of PCV13 in this population.” [26–28].

The burden of CAP among VHA patients was especially high among those aged ≥50 years and among those who had underlying chronic medical or immunocompromising conditions. From the VHA perspective, incidence of CAP among those aged 50–64 years was comparable to those over age 65. This observation, however, must be interpreted in the context of the VHA population. Namely, that nearly all patients aged ≥65 years are dually eligible for Medicare insurance. Therefore, some elderly VHA patients may seek care for CAP outside of the VHA system with health insurance coverage provided by Medicare and, subsequently, would not have been captured in our analysis.

In addition to age, risk status was also related to CAP burden. Specifically, compared to those at low risk, moderate- and high-risk patients were >3 and >6 times more likely to develop CAP, respectively. A more granular look at specific comorbid illnesses revealed that, compared to patients without comorbid or immunocompromising conditions, patients with a history of diabetes, dementia, or chronic heart, cerebrovascular, lung, or liver disease were 3–14 times more likely to develop CAP. This is especially worth noting, given that half of VHA patients aged ≥50 years and nearly two-thirds of VHA patients aged ≥65 years had ≥1 chronic medical or immunocompromising condition. Taken together, these data underscore the importance of CAP prevention and the management of modifiable risk factors among a subpopulation at especially high risk of developing CAP. Indeed, not only did nearly two-thirds of all patients eligible for VHA benefits aged ≥65 years have ≥1 chronic medical or immunocompromising conditions, but of those who developed CAP, 91 % had ≥1 chronic medical or immunocompromising condition. The most prevalent chronic (non-immunocompromising) medical conditions among those who developed CAP included history of heart failure, coronary artery disease, diabetes, and COPD. Although not common among those aged ≥65 years, among those aged 50–64 years, chronic liver disease and alcoholism were prevalent among those who developed CAP in the VHA. Immunocompromising conditions were composed mainly of chronic renal disease or solid organ cancer. Although not typically included in the list of conditions thought to lead to increased risk of CAP [19–21], a history of either stroke or dementia lead to >5 times increased risk among those aged 50–64 years and >7 times increased risk among those aged ≥65 years compared to patients without comorbid or immunocompromising conditions.

In addition to risk status predicting the development of CAP, risk status—independent of age and other demographic characteristics—was strongly associated with rates of hospitalization, 30-day readmission (among those who were hospitalized), and death. Thus, not only were patients with underlying comorbid or immunocompromising conditions more likely to develop CAP, but also, if they did ultimately develop CAP, moderate- and high-risk patients were more likely to be hospitalized and had higher mortality rates. For example, results from this study showed that, among all adult patients eligible for VHA care, only 21 % of low-risk patients were hospitalized for CAP, however, that number climbed to 45 % hospitalized among moderate-risk patients and 56 % hospitalized among high-risk patients. Same year, all-cause mortality rates showed a similar trend, with 7, 14, and 32 % of low-, moderate-, and high-risk patients, respectively, dying in the same year they developed CAP. Patients with underlying comorbid or immunocompromising conditions also had higher health care costs. Compared to low-risk patients, median (90-day) CAP episode costs for moderate and high-risk patients were $901 (95 %CI $461, $1,341) and $4,659 (95 %CI $4,182, $5,135) higher, respectively.

Previous studies have suggested that, compared to the general US population or Veterans who do not use the VHA system, VHA patients report poorer physical and mental health and have more underlying chronic health conditions. [15, 16] Results from this study confirm these findings, as data from our report showed that a higher proportion of VHA patients had underlying immunocompromising or chronic medical conditions compared to the general privately insured population [21]. These results, combined with the fact that patients at moderate or high risk for developing CAP were more likely to be hospitalized and had higher mortality rates, suggest that a focus on CAP prevention and the management of modifiable risk factors among those with comorbid or immunocompromising conditions may be especially important in the VHA system. These data are compounded by evidence, both in this study and elsewhere [14], demonstrating that the incidence of CAP is related to being both older and male—characteristics typical of the Veteran population.

## Limitations

This study was not without limitations. Primarily, the manner in which we defined an episode of CAP was based on an algorithm of diagnostic and procedural claims. While this definition has been used before [21, 29, 30], diagnostic codes are not always accurate. Specifically, we could only assess whether a chest X-ray was performed within 2 weeks of diagnosis—whether or not the chest X-ray was conclusive of pneumonia was unknown. Another limitation is the inability to always accurately distinguish between community-acquired and health care-associated pneumonia. Our definition of *community*-*acquired* pneumonia excluded patients that had any inpatient encounters in the prior 90 days. This definition is likely conservative and may underestimate the incidence of CAP, as other studies have used 7-day [29] or 14-day [30] windows (rather than 90 days) to distinguish between community-acquired and health care-associated pneumonia. Finally, although this study captured the burden of CAP from the perspective of the VHA—it likely underestimated the true burden of CAP for all VHA patients. Specifically, given that many VHA patients (especially those aged ≥65 years who are eligible for Medicare) are dually eligible for supplemental insurance, it is likely that some VHA patients sought care for CAP outside of the VHA system and would not have been captured in our analysis [31–34]. As such, our incidence rates should be interpreted with caution and only in this context. In the same vein, it is also possible; however, that we classified some health care-associated cases of pneumonia as *community*-*acquired*—where prior hospitalization or long-term did occur in the 90 days prior to a pneumonia diagnosis, but it occurred in a non-VHA setting.

## Conclusion

In 2011, >35,000 CAP episodes occurred in the VHA (18,375 CAP episodes among those aged ≥65 years), despite historically high coverage with pneumococcal polysaccharide vaccine (>80 %). [24] Economic data revealed that CAP episodes were associated with a notable increase in cost during the 90-day CAP episode period and could account for as much as $750M ($415M for those aged ≥65 years) in additional annual medical costs—the large majority of which ($720M; 96 %) were due to additional expenditures related to hospitalization and other inpatient services. Moreover, the true burden among VHA patients was likely higher—perhaps considerably so—given that many patients have supplemental insurance (namely Medicare) and may have sought treatment for CAP outside of the VHA system. In addition, more than half of all VHA patients aged ≥50 and nearly two-thirds of all VHA patients aged ≥65 years had ≥1 chronic medical or immunocompromising conditions, and these conditions were associated with several fold higher risk of developing CAP. In summary, a renewed focus on CAP prevention and the management of modifiable risk factors in the VHA is important. Prevention is especially important among older patients and patients with underlying comorbid or immunocompromising conditions, as these patients were not only more likely to develop CAP, but were also more costly and more likely to be hospitalized, readmitted within 30 days, and die if they did develop CAP.

## Electronic supplementary material

Supplementary material 1 (DOCX 75 kb)
