# Supplementary material for: Clinical and economic burden of community-acquired pneumonia in the Veterans Health Administration, 2011: a retrospective cohort study
Source: Infection. 2015 May 17;43(6):671–80. doi: 10.1007/s15010-015-0789-3 (PMC4656694; doi:10.1007/s15010-015-0789-3)
Supplement: Supplementary file 1 — Supplementary material 1 (DOCX 75 kb) [file 15010_2015_789_MOESM1_ESM.docx]

Supplementary Online Content

eTable 1 Sociodemographic Characteristics of Veterans Health Administration-eligible
 Persons who Developed Community-acquired Pneumonia, 2011 (n=34,101)

eTable 2 Clinical Characteristics of Veterans Health Administration-eligible Persons who
 Developed Community-acquired Pneumonia (CAP), 2011 (n=34,101)

eTable 3 Predictors of Hospital Admission, 30-day Readmission, and Death among Veterans Health Administration-eligible Persons who Developed Community-acquired Pneumonia (CAP), 2011 (n=34,101)

eTable 4 Calculating the Average Excess Economic Burden of Community-acquired Pneumonia (CAP) among Veterans Health Administration-eligible Persons, 2011 (n=34,101)

eTable 5 Quantile Regression Modeling Estimating the Impact of Select Risk Factors on Median 90-day Community-acquired Pneumonia Episode Cost among Patients in the Veterans Health Administration, 2011 (n=34,049)

eFigure 1 Average Per-patient, Per-month (PPPM) Costs Before, During, and After the Development of Community-acquired Pneumonia (CAP) among Veterans Health Administration-eligible Persons who CAP by Age and Risk Status, 2011 (n=34,049)

This supplementary material has been provided by the authors to give readers additional information about their work.

eTable 1 Sociodemographic Characteristics of Veterans Health Administration-eligible
 Persons who Developed Community-acquired Pneumonia, 2011 (n=34,101)

| Characteristic | n | % |
| --- | --- | --- |
| Age |  |  |
| 18-49 | 3,557 | 10 |
| 50-64 | 12,962 | 38 |
| 65-79 | 10,515 | 31 |
| ≥80 | 7,067 | 21 |
| Gender |  |  |
| Male | 32,322 | 95 |
| Female | 1,779 | 5 |
| Race |  |  |
| White | 25,312 | 74 |
| Black or African American | 5,243 | 15 |
| Asian | 324 | 1 |
| American Indian or Alaskan Native | 147 | <1 |
| Mixed Race | 631 | 2 |
| Unknown | 2,444 | 7 |
| Ethnicity |  |  |
| Hispanic | 941 | 3 |
| Non-Hispanic | 33,160 | 97 |
| Marital Status |  |  |
| Married | 15,960 | 47 |
| Divorced or Separated | 10,499 | 31 |
| Widowed | 3,643 | 11 |
| Never Married | 3,834 | 11 |
| Unknown | 165 | <1 |
| Geographic Region* |  |  |
| Northeast | 5,063 | 15 |
| Southeast | 10,760 | 32 |
| Midwest | 7,226 | 21 |
| Southwest | 4,515 | 13 |
| West | 6,037 | 18 |
| Living outside of the United States | 501 | 1 |
| VA Eligibility |  |  |
| Service Connected 50-100% | 9,094 | 27 |
| Service Connected <50% | 6,170 | 18 |
| Not Service Connected | 17,263 | 51 |
| CHAMPVA^‡^ | 135 | <1 |
| Other^†^ | 1,439 | 4 |
| Copayment Status |  |  |
| Exempt | 27,971 | 82 |
| Required | 6,130 | 18 |
| Supplemental Insurance |  |  |
| Yes | 21,448 | 63 |
| No | 12,524 | 37 |
| Unknown | 129 | <1 |

*Northeast: Connecticut, Delaware, District of Columbia, Maine, Maryland, Massachusetts, New Hampshire, New Jersey, New York, Pennsylvania, Rhode Island, Vermont. Southeast: Alabama, Arkansas, Florida, Georgia, Kentucky, Louisiana, Mississippi, North Carolina, South Carolina, Tennessee, Virginia, West Virginia. Midwest: Illinois, Indiana, Iowa, Kansas, Michigan, Missouri, Minnesota, Nebraska, North Dakota, Ohio, South Dakota, Wisconsin. Southwest: Arizona, New Mexico, Oklahoma, Texas. West: Alaska, California, Colorado, Hawaii, Idaho, Montana, Nevada, Oregon, Utah, Washington, Wyoming.

^‡^CHAMPVA is the Civilian Health and Medical Program of the Department of Veterans Affairs (CHAMPVA) and is a comprehensive health care program in which the VA shares the cost of covered health care services and supplies with eligible beneficiaries. To be eligible for CHAMPVA, you cannot be eligible for TRICARE/CHAMPUS and you must be in one of these categories: i) the spouse or child of a veteran who has been rated permanently and totally disabled for a service-connected disability by a VA regional office, or ii) the surviving spouse or child of a veteran who died from a VA-rated service-connected disability, or iii) the surviving spouse or child of a veteran who was at the time death rated permanently and totally disabled, or iv) the surviving spouse or child of a military member who died in the line of duty, not due to misconduct (in most of these cases, these family members are eligible for TRICARE, not CHAMPVA).

^†^Other includes: Active duty, aid & attendance, allied veteran, DOD dependent, retiree dependent, employee, housebound individuals, humanitarian emergencies, military retiree, prisoner of war, Purple Heart recipients, TRICARE, and other federal agencies.

eTable 2 Clinical Characteristics of Veterans Health Administration-eligible Persons who
 Developed Community-acquired Pneumonia (CAP), 2011 (n=34,101)

| Clinical Characteristic / Comorbidity | 18-49 years  (n=3,557) | | 50-64 years  (n=12,962) | | 65-79 years  (n=10,515) | | ≥80 years  (n=7,067) | |
| --- | --- | --- | --- | --- | --- | --- | --- | --- |
|  | *n, %* | | | | | | | |
| *Aggregate Risk for Developing CAP** |  |  |  |  |  |  |  |  |
| High / Immunocompromised | 379 | 11 | 3,694 | 28 | 4,291 | 41 | 3,195 | 45 |
| Moderate / At-risk | 1,193 | 34 | 7,231 | 56 | 5,313 | 51 | 3,208 | 45 |
| Low | 1,985 | 56 | 2,037 | 16 | 911 | 9 | 664 | 9 |
| *High Risk / Immunocompromising Conditions* |  |  |  |  |  |  |  |  |
| HIV | 102 | 3 | 279 | 2 | 63 | <1 | 7 | <1 |
| Lymphoma | 28 | <1 | 193 | 1 | 184 | 2 | 107 | 2 |
| Myeloma | 1 | <1 | 57 | <1 | 61 | <1 | 45 | <1 |
| Leukemia | 17 | <1 | 126 | <1 | 170 | 2 | 113 | 2 |
| Solid Organ Cancer | 163 | 5 | 2,444 | 19 | 3,131 | 30 | 2,285 | 32 |
| Nephrotic Syndrome | 10 | <1 | 88 | <1 | 59 | <1 | 24 | <1 |
| Chronic Renal Disease | 95 | 3 | 991 | 8 | 1,412 | 13 | 1,249 | 18 |
| Transplantation | 19 | <1 | 154 | 1 | 105 | 1 | 15 | <1 |
| Asplenic | 10 | <1 | 74 | <1 | 43 | <1 | 41 | <1 |
| Cochlear Implant | 1 | <1 | 2 | <1 | 11 | <1 | 4 | <1 |
| Cerobrospinal Fluid Leak | 0 | 0 | 3 | <1 | 3 | <1 | 1 | <1 |
| *Moderate / At-risk Conditions* |  |  |  |  |  |  |  |  |
| Heart Failure | 114 | 3 | 2,132 | 16 | 2,789 | 27 | 2,573 | 36 |
| Coronary Artery Disease | 182 | 5 | 3,784 | 29 | 4,837 | 46 | 3,718 | 53 |
| Cardiomyopathy | 62 | 2 | 780 | 6 | 796 | 8 | 508 | 7 |
| Diabetes Mellitus | 382 | 11 | 4,590 | 35 | 4,587 | 44 | 2,570 | 36 |
| Asthma | 481 | 14 | 1,600 | 12 | 1,103 | 10 | 590 | 8 |
| Chronic Obstructive Pulmonary Disorder | 304 | 9 | 5,420 | 42 | 5,544 | 53 | 3,187 | 45 |
| Chronic Liver Disease | 147 | 4 | 1,906 | 15 | 580 | 6 | 156 | 2 |
| Alcoholism | 483 | 14 | 2,906 | 22 | 1,143 | 11 | 227 | 3 |
| *Other Conditions* |  |  |  |  |  |  |  |  |
| Dementia | 53 | 1 | 407 | 3 | 942 | 9 | 2,030 | 29 |
| Stroke | 54 | 2 | 1,179 | 9 | 1,789 | 17 | 1,658 | 23 |

*Low risk was defined as immunocompetent without chronic medical conditions. Moderate risk was defined as immunocompetent with ≥1 chronic medical condition. High risk was defined as immunocompromised.

eTable 3 Predictors of Hospital Admission, 30-day Readmission, and Death among Veterans Health Administration-eligible Persons who Developed Community-acquired Pneumonia (CAP), 2011 (n=34,101)

|  | Hospitalization n=34,101 | | 30-day Readmission n=15,265^#^ | | Death in Year of CAP n=34,101 | |
| --- | --- | --- | --- | --- | --- | --- |
| Characteristic | Est. RR^‡^ | 95%CI | OR | 95%CI | OR | 95%CI |
| Age (years) |  |  |  |  |  |  |
| 18-49 | 1.00 |  | 1.00 |  | 1.00 |  |
| 50-64 | 1.64 | 1.53, 1.76 | 1.15 | 0.91, 1.45 | 3.86 | 3.09, 4.82 |
| 65-79 | 1.84 | 1.71, 1.98 | 1.15 | 0.90, 1.47 | 11.5 | 9.12, 14.4 |
| ≥80 | 2.11 | 1.96, 2.27 | 1.00 | 0.77, 1.28 | 23.8 | 18.9, 29.9 |
| Gender |  |  |  |  |  |  |
| Female | 1.00 |  | 1.00 |  | 1.00 |  |
| Male | 1.20 | 1.12, 1.29 | 1.62 | 1.20, 2.20 | 1.39 | 1.15, 1.69 |
| Race |  |  |  |  |  |  |
| White | 1.00 |  | 1.00 |  | 1.00 |  |
| Black or African American | 1.00 | 0.97, 1.07 | 0.93 | 0.82, 1.06 | 0.90 | 0.82, 0.98 |
| Asian | 0.81 | 0.69, 0.95 | 1.08 | 0.62, 1.88 | 0.82 | 0.58, 1.17 |
| Am. Indian or Alaskan Native | 0.83 | 0.67, 1.03 | 1.00 | 0.47, 2.16 | 0.80 | 0.46, 1.39 |
| Mixed Race | 1.00 | 0.93, 1.08 | 1.08 | 0.80, 1.47 | 0.90 | 0.72, 1.13 |
| Unknown | 0.93 | 0.88, 0.98 | 0.91 | 0.75, 1.10 | 1.15 | 1.02, 1.30 |
| Ethnicity |  |  |  |  |  |  |
| Non-Hispanic | 1.00 |  | 1.00 |  | 1.00 |  |
| Hispanic | 1.11 | 1.04, 1.19 | 1.06 | 0.78, 1.44 | 1.01 | 0.82, 1.25 |
| Marital Status |  |  |  |  |  |  |
| Unmarried | 1.00 |  | 1.00 |  | 1.00 |  |
| Married | 0.90 | 0.88, 0.92 | 0.95 | 0.87, 1.04 | 0.99 | 0.93, 1.06 |
| Geographic Region* |  |  |  |  |  |  |
| Northeast | 1.00 |  | 1.00 |  | 1.00 |  |
| Southeast | 0.99 | 0.96, 1.02 | 0.99 | 0.86, 1.13 | 1.19 | 1.09, 1.31 |
| Midwest | 0.99 | 0.95, 1.02 | 1.11 | 0.96, 1.28 | 0.99 | 0.90, 1.10 |
| Southwest | 1.02 | 0.98, 1.06 | 0.95 | 0.81, 1.12 | 1.14 | 1.02, 1.27 |
| West | 0.89 | 0.86, 0.93 | 0.79 | 0.67, 0.93 | 0.88 | 0.79, 0.98 |
| Lived outside of the US | 1.08 | 1.00, 1.16 | 0.90 | 0.63, 1.29 | 1.19 | 0.95, 1.49 |
| VA Eligibility |  |  |  |  |  |  |
| Service Connected 50-100% | 1.00 |  | 1.00 |  | 1.00 |  |
| Service Connected <50% | 0.98 | 0.94, 1.01 | 0.94 | 0.82, 1.08 | 1.19 | 1.09, 1.31 |
| Not Service Connected | 1.04 | 1.01, 1.07 | 0.91 | 0.82, 1.01 | 1.11 | 1.03, 1.20 |
| CHAMPVA | 0.77 | 0.56, 1.05 | 0.98 | 0.29, 3.37 | 0.35 | 0.08, 1.47 |
| Other^†^ | 1.10 | 1.04, 1.16 | 1.00 | 0.80, 1.26 | 1.39 | 1.20, 1.62 |
| Supplemental Insurance |  |  |  |  |  |  |
| Yes | 1.00 |  | 1.00 |  | 1.00 |  |
| No | 1.13 | 1.10, 1.16 | 1.21 | 1.09, 1.34 | 3.66 | 3.38, 3.95 |
| Unknown | 0.92 | 0.71, 1.18 | 1.53 | 0.70, 3.37 | 0.78 | 0.37, 1.65 |
| Copayment Status |  |  |  |  |  |  |
| Exempt | 1.00 |  | 1.00 |  | 1.00 |  |
| Required | 0.82 | 0.79, 0.85 | 0.92 | 0.80, 1.05 | 1.01 | 0.93, 1.10 |
| Aggregate Risk of CAP^§^ |  |  |  |  |  |  |
| Low | 1.00 |  | 1.00 |  | 1.00 |  |
| Moderate / At-risk | 1.85 | 1.71, 1.98 | 1.47 | 1.19, 1.80 | 1.51 | 1.34, 1.71 |
| High / Immunocompromised | 2.11 | 1.96, 2.27 | 1.90 | 1.54, 2.33 | 3.75 | 3.32, 4.23 |
| Hospitalized due to CAP |  |  |  |  |  |  |
| No | *n.a.* | *n.a.* | *n.a.* | *n.a.* | 1.00 |  |
| Yes | *n.a.* | *n.a.* | *n.a.* | *n.a.* | 2.22 | 2.09, 2.36 |

^#^The model analyzing predictors of 30-day readmission only included patients who were hospitalized.

*Northeast: Connecticut, Delaware, District of Columbia, Maine, Maryland, Massachusetts, New Hampshire, New Jersey, New York, Pennsylvania, Rhode Island, Vermont. Southeast: Alabama, Arkansas, Florida, Georgia, Kentucky, Louisiana, Mississippi, North Carolina, South Carolina, Tennessee, Virginia, West Virginia. Midwest: Illinois, Indiana, Iowa, Kansas, Michigan, Missouri, Minnesota, Nebraska, North Dakota, Ohio, South Dakota, Wisconsin. Southwest: Arizona, New Mexico, Oklahoma, Texas. West: Alaska, California, Colorado, Hawaii, Idaho, Montana, Nevada, Oregon, Utah, Washington, Wyoming

^†^Other includes: Active duty, aid & attendance, allied veteran, DOD dependent, retiree dependent, employee, housebound individuals, humanitarian emergencies, military retiree, prisoner of war, Purple Heart recipients, Tricare, and other federal agencies.

^§^Low risk was defined as immunocompetent without chronic medical conditions. Moderate risk was defined as immunocompetent with ≥1 chronic medical condition. High risk was defined as immunocompromised.

^‡^Relative risk estimated from a log binomial model because hospitalization event was not rare (45%).

eTable 4 Calculating the Average Excess Economic Burden of Community-acquired Pneumonia (CAP) among Veterans Health Administration-eligible Persons, 2011 (n=34,101)*

*52 of 34,101 patients were missing valid economic data from the HERC dataset. Thus, economic estimates were based on 34,049 VHA patients. Data were adjusted to account for local variations in costs.

eTable 5 Quantile Regression Modeling Estimating the Impact of Select Risk Factors on Median 90-day Community-acquired Pneumonia Episode Cost among Patients in the Veterans Health Administration, 2011 (n=34,049)*

| Characteristic | Coefficient^‡^ | 95%CI | p-value |
| --- | --- | --- | --- |
| Intercept | 4,233 | 2,517, 5.950 | <.001 |
| Age (years) |  |  |  |
| 18-49 | reference |  |  |
| 50-64 | 794 | 256, 1,333 | <.01 |
| 65-79 | 1,047 | 445, 1,651 | <.01 |
| ≥80 | 654 | 22, 1,287 | .04 |
| Gender |  |  |  |
| Female | reference |  |  |
| Male | 267 | -421, 954 | .45 |
| Race |  |  |  |
| White | reference |  |  |
| Black or African American | 445 | 35, 855 | .03 |
| Asian | -189 | -1,702, 1,324 | .80 |
| Am. Indian or Alaskan Native | -357 | -2,557, 1,844 | .75 |
| Mixed Race | 130 | -941, 1,201 | .81 |
| Unknown | -496 | -1,061, 69 | .09 |
| Ethnicity |  |  |  |
| Non-Hispanic | reference |  |  |
| Hispanic | 89 | -987, 1,166 | .87 |
| Marital Status |  |  |  |
| Unmarried | reference |  |  |
| Married | -574 | -875, -273 | <.001 |
| VA Eligibility |  |  |  |
| Service Connected 50-100% | reference |  |  |
| Service Connected <50% | -681 | -1,121, -241 | <.01 |
| Not Service Connected | -389 | -761, -16 | .04 |
| CHAMPVA | 30 | -2,383, 2,442 | .98 |
| Other^†^ | 812 | 42, 1,584 | .04 |
| Supplemental Insurance |  |  |  |
| Yes | reference |  |  |
| No | 586 | 236, 937 | <.01 |
| Unknown | -456 | -2,856, 1,943 | .71 |
| Copayment Status |  |  |  |
| Exempt | reference |  |  |
| Required | -651 | -1,059, -242 | .002 |
| Aggregate Risk of CAP^§^ |  |  |  |
| Low | reference |  |  |
| Moderate / At-risk | 901 | 461, 1,341 | <.001 |
| High / Immunocompromised | 4,659 | 4,182, 5,135 | <.001 |
| Hospitalized due to CAP |  |  |  |
| No | reference |  |  |
| Yes | 15,101 | 14,710, 15,311 | <.001 |

*52 of 34,101 patients were missing valid economic data from the HERC dataset. Thus, economic estimates were based on 34,049 VHA patients. Data were adjusted to account for local variations in costs.

^†^Other includes: Active duty, aid & attendance, allied veteran, DOD dependent, retiree dependent, employee, housebound individuals, humanitarian emergencies, military retiree, prisoner of war, Purple Heart recipients, Tricare, and other federal agencies.

^§^Low risk was defined as immunocompetent without chronic medical conditions. Moderate risk was defined as immunocompetent with ≥1 chronic medical condition. High risk was defined as immunocompromised.

^‡^The coefficient is the estimated median increase compared to the reference category from non-parametric quantile regression modeling.

eFigure 1 Average Per-patient, Per-month (PPPM) Costs Before, During, and After the Development of Community-acquired Pneumonia (CAP) among Veterans Health Administration-eligible Persons who CAP by Age and Risk Status^†^, 2011 (n=34,049)*

^§^Low risk was defined as immunocompetent without chronic medical conditions. Moderate risk was defined as immunocompetent with ≥1 chronic medical condition. High risk was defined as immunocompromised.

*52 of 34,101 patients were missing valid economic data from the HERC dataset. Data were adjusted to account for local variations in costs.
